# In-Field Nondestructive Detection of Nitrogen Status on ‘Yotsuboshi’ Strawberry Using Deep Learning Algorithm

**DOI:** 10.3390/s26103107

**Published:** 2026-05-14

**Authors:** Bryan V. Apacionado, Tofael Ahamed

**Affiliations:** 1 Graduate School of Science and Technology, University of Tsukuba, 1-1-1 Tennodai, Tsukuba 305-8577, Japan; s2330268@u.tsukuba.ac.jp; 2 Institute of Crop Science, College of Agriculture and Food Science, University of the Philippines Los Baños, Laguna 4031, Philippines; 3 Institute of Life and Environmental Sciences, University of Tsukuba, 1-1-1 Tennodai, Tsukuba 305-8577, Japan

**Keywords:** nondestructive, nitrogen status, deep learning, strawberry

## Abstract

Nitrogen (N) management is critical for optimizing growth and fruit quality in open-field strawberry cultivation, demanding advanced technological solutions for reliable nutrient assessment. However, visual symptom diagnosis, though widely utilized for nutrient monitoring, is inherently subjective and prone to observer bias, resulting in inconsistent and often unreliable assessments. While available accurate tissue analysis is destructive and costly. Nondestructive, in-field imaging techniques such as the normalized difference vegetation index (NDVI) exist but require expensive multispectral imaging systems. To address these limitations, this study developed a streamlined methodology for in-field N status detection using deep learning on standard RGB images. The experiment utilized ‘Yotsuboshi’ strawberries in a randomized complete block design with sufficient nitrogen (T1) and deficient nitrogen (T2) treatments. To mitigate ambient light variability, a key challenge in open-field phenotyping, a low-cost phenotyping cylinder was developed for standardized smartphone image acquisition. Rigorous four-stage annotation criteria were also introduced to classify the nitrogen status in strawberry leaves as NormalN, LowN, or AdvancedLowN, ensuring a high-quality novel dataset. A YOLO11 model trained on this dataset achieved precision, recall, and mAP50 values exceeding 99%. Subsequent testing using the phenotyping cylinder yielded a mAP50 of 87%. In-field validation without a phenotyping cylinder also demonstrated robust performance under diffuse cloudy conditions (82.7% mAP50), outperforming direct sunlight (79% mAP50). Moreover, the model’s classifications of ‘NormalN’ and ‘LowN’ statuses strongly corresponded with NDVI measurements, validating the accuracy of the RGB-based approach. This research demonstrates the significant potential of combining deep learning and phenotyping cylinder to create a rapid, low-cost, nondestructive and reliable tool for in-field nitrogen detection, with possible application across different crops and environmental conditions.

## 1. Introduction

Strawberry (*Fragaria* × *ananassa* Duch.) is a globally significant horticultural crop and is considered one of the most cultivated small fruits because of its distinct sensory characteristics and nutritional benefits [1,2]. However, in terms of cultivation, it is a notably sensitive crop, requiring precise nutrient management to achieve high yields and superior fruit quality. Maintaining an optimal nutritional status is therefore crucial for both successful production and overall food safety [3]. Nitrogen (N) stands out as one of the most essential nutrients for successful strawberry production that plays a pivotal role in critical developmental processes, including vegetative growth, runner production, and fruit bud formation [3,4,5]. Deficiencies in this vital nutrient result in clear visual symptoms in strawberry plants. The initial symptoms of nitrogen deficiency first occur on older, mature leaves. This phenomenon is a direct result of the high mobility of nitrogen within the plant, which enables its translocation from older tissues to support new, developing growth [6]. These older leaves initially appear smaller and exhibit pale green or yellow chlorosis. As the deficiency progresses, this chlorosis becomes more uniform across the leaf, and in advanced stages, the blades and petioles may develop a reddish or purplish pigmentation [7]. These symptoms are a direct result of inhibited chlorophyll synthesis, which is essential for photosynthesis [8]. Early detection of nitrogen deficiency is essential to prevent stunted growth and yield loss in strawberries. Timely identification allows for corrective interventions that safeguard both fruit quality and overall productivity of the strawberry.

While the effects of nitrogen are extensively documented across various crops, the advancement of precision agriculture necessitates variety-specific research to optimize management for emerging cultivars. A comprehensive study on strawberry leaf analysis demonstrates that nutrient concentrations and diagnostic baselines exhibit significant variation among different cultivars [9]. Therefore, establishing variety-specific evaluation parameters is essential for developing precise, effective nutrient management strategies tailored to the unique physiological needs of a given strawberry variety. The 2017 release of the Japanese strawberry ‘Yotsuboshi’ creates a critical need for such research to fully understand its unique growth and developmental patterns. A dedicated study on nitrogen status detection is vital given that ‘Yotsuboshi’ possesses distinct biological, physiological, and commercial characteristics, most notably its seed-propagating F1 hybrid nature and superior organoleptic profile, that set it apart from traditional cultivars [10,11].

Traditionally, the assessment of nitrogen status in strawberry plants has relied on methods such as visual inspection and laboratory-based chemical analysis. While visual assessment in strawberry is immediate, it is subjective and can be unreliable. More precise methods, such as the conventional Kjeldahl digestion and modern Dumas combustion, provide accurate measurements of nitrogen content in strawberry plant tissues but are inherently destructive, costly, and time-consuming and require specialized equipment and expertise, making them impractical for frequent, large-scale monitoring [12,13,14]. To overcome the limitations of these methods, recent research focused on developing nondestructive, rapid, and accurate techniques for nutrient monitoring in strawberry. There is a growing body of work on the use of vegetation indices to detect nutrient deficiencies and other stresses in strawberry plants [15]. Among the most common is the normalized difference vegetation index (NDVI), a quantitative metric used to assess plant health and density [16]. The NDVI is calculated from the differential reflectance of red and near-infrared (NIR) light by the strawberry plant canopy, providing a reliable measure of vegetation vigor [17]. Although the NDVI does not directly measure the nitrogen content of the strawberry, a strong positive correlation has been established between the NDVI values and the chlorophyll content and overall biomass. Since these parameters are physiologically linked to the nitrogen status within the plant, the NDVI can function as an effective proxy and a potential screening tool for diagnosing nitrogen deficiency [18,19]. However, for many strawberry growers, the use of the NDVI presents significant practical challenges in the detection of nitrogen status owing to the cost, equipment required, and complexity of the process. This method is financially prohibitive, as it necessitates substantial investment in specialized multispectral cameras and drones [20]. Moreover, it is technically complex and requires expertise in equipment operation, calibration, and sophisticated data processing. Finally, the NDVI is not a direct indicator of nitrogen, as it reflects general plant health and can be confounded by other stressors, making accurate interpretation difficult without specialized knowledge [16].

A more cost-effective and accessible alternative is the use of RGB imaging, which recent studies have successfully employed to estimate nitrogen levels across various crops. A study on basil using RGB images demonstrated that among various evaluated indices, the Normalized Green-Red Difference Index (NGRDI), Red Index (RI), and Color Index of Vegetation Extraction (CIVE) were the most effective indicators of nitrogen concentration, showing exceptionally strong correlations (r = 0.92–0.93) [21]. Another study found that high-definition RGB images from UAVs can effectively monitor winter wheat nitrogen status [22], with image textures providing more accurate estimations than standard vegetation indices. Specifically, Gabor-based textures in a GPR model accurately estimated winter wheat N density (PND) (R^2^_val_ = 0.675, RMSE_val_ = 2.493 g/m^2^), while GLCM-based textures in a PLSR model best predicted plant N concentration (PNC) (R^2^_val_ = 0.612, RMSE_val_ = 0.380). Meanwhile, for coffee, research proposed a method to standardize RGB camera index values using exposure value (EV) and Hue adjustment to overcome the challenges of inconsistent natural light and shading in coffee plantations [23]. By calibrating digital numbers, this approach enables a low-cost, accurate, and non-destructive way for farmers in developing countries to monitor plant nitrogen and greenness throughout the day. A study on maize using a custom field robot equipped with an RGB camera demonstrated that under-canopy imaging of maize can accurately detect nitrogen stress by capturing spectral data from lower canopy levels [24]. The method achieved high precision with R^2^ values of 0.78 during the day and 0.92 at night, while semivariogram analysis revealed that nitrogen stress patterns are most distinct at a spatial range of less than 6 m.

In recent years, deep learning has emerged as a powerful tool for monitoring plant nutrient status, offsetting the inherent limitations of RGB imaging by eliminating complex preprocessing steps and simplifying practical applications. However, its implementation remains largely confined to controlled environments due to the significant challenges posed by variable ambient lighting, which often degrades model performance under field conditions [25]. For instance, a study compared ResNet-50 and a custom CNN for classifying strawberry nitrogen levels across three fertilization treatments using digitized RGB images, with ResNet-50 achieving significantly higher accuracy (78% vs. 48%). However, a primary limitation was the reliance on the destructive collection of leaf samples and flatbed scanner digitization within a controlled environment; while this provides high-quality images, it lacks the real-time efficiency required for field-based monitoring [26].

Bridging this gap between laboratory accuracy and field applicability remains a core challenge in precision agriculture. Recent advancements have shown that modern architectures can handle these complexities; for example, a study successfully identified nitrogen deficiency in maize by applying deep learning to field-based RGB images, comparing a custom vision transformer against various fine-tuned CNN models [27]. The results demonstrated that while vision transformers showed potential, a fine-tuned EfficientNetB0 model proved most effective for practical use, achieving a maximum classification accuracy of 97% across three stress levels.

Despite progress in other crops, machine vision applications in strawberry cultivation have largely relied on datasets collected in controlled environments and have been focused on tasks like defect identification or quality evaluation [28,29]. Consequently, a significant gap remains between academic research and practical field adoption. Many advanced machine vision technologies remain underutilized on farms because they are often expensive, operationally complex, and lack user-friendly interfaces for farm managers [15,30]. To address these challenges, this study aimed to develop a nondestructive, variety-specific detection model for nitrogen status monitoring in ‘Yotsuboshi’ strawberry plants using a custom-built phenotyping cylinder and a deep learning algorithm anchored on a rigorous four-stage annotation criterion to ensure a high-fidelity dataset that can offer a promising, accessible, and efficient tool for precision agriculture.

## 2. Materials and Methods

**Figure 1:**
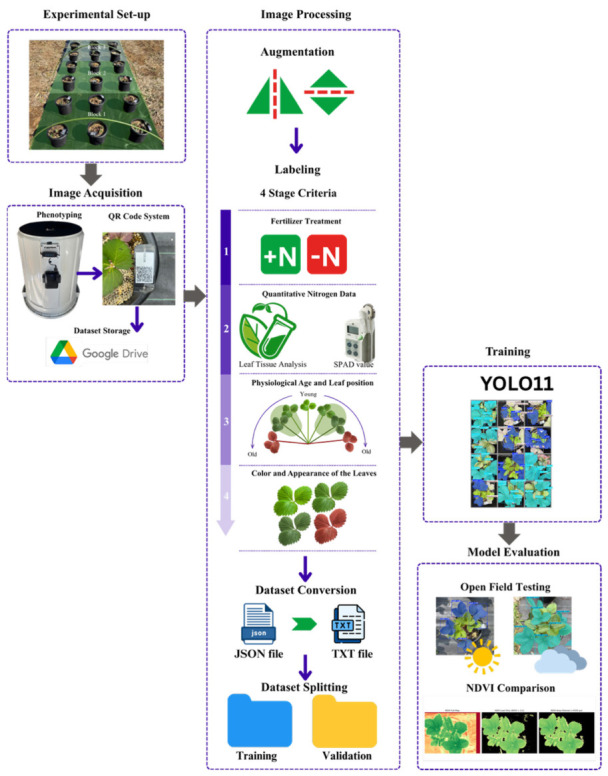
Conceptual framework for detecting the nitrogen status of ‘Yotsuboshi’ strawberry.

This section details the research methodology used to determine the nitrogen status of ‘Yotsuboshi’ strawberry plants under open-field conditions (Figure 1).

### 2.1. Plant Material and Cultivation Conditions

**Figure 2:**
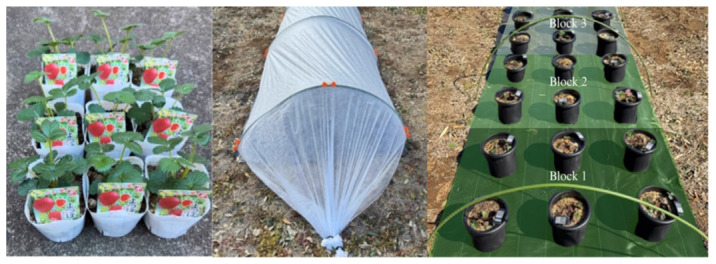
Experimental setup of ‘Yotsuboshi’ strawberry plants at the Tsukuba-Plant Innovation Research Center (T-PIRC), University of Tsukuba, Japan.

This study was conducted on strawberry plants (*Fragaria* × *ananassa* ‘Yotsuboshi’) from April to July 2025 under open-field conditions at the Tsukuba-Plant Innovation Research Center (T-PIRC), University of Tsukuba, Japan (Figure 2).

It utilized a cultivar called ‘Yotsuboshi’ that differs from traditional varieties in its reproductive and developmental strategies, which directly influence how nitrogen is utilized and detected. Unlike traditional cultivars that are propagated vegetatively through runners, ‘Yotsuboshi’ is grown from seeds, resulting in independent seedlings that exhibit different early-stage nutrient requirements. While runner-based plants can rely on a mother plant for initial nutrients, these seed-based seedlings must establish their own vigor, making precisely calibrated nitrogen monitoring essential for their success in high-density, year-round production systems. Considering the high seedling density requirement of strawberry cultivation (approximately 7000 seedlings/100 m^2^), the conventional reliance on runners is a significant bottleneck. This vegetative process is labor-intensive, yielding only 20 to 40 seedlings per mother plant and necessitating a complex, multitiered supply chain or obligating farmers to propagate their own stock. In contrast, seed-based propagation allows for centralized, commercial-scale production by specialized companies, boasting a propagation efficiency that is more than 40 times greater than that of vegetative methods. Reflecting its commercial appeal, the cultivation area for this variety has expanded significantly in Japan, increasing by 177% from 2018 to 2022 [10].

The plants (2-month-old) were purchased, transferred and cultivated for another 4 months in 8 L pots with a low-nutrient substrate composed of equal parts garden soil, pumice, vermiculite, perlite, and kanuma soil. Throughout the experiment, the plants were housed within a protective net tunnel to protect them from pests and received uniform irrigation. To deliberately induce nitrogen deficiency for the study, no fertilizer was applied to any of the plants for one month prior to the application of the treatment. In addition, to maintain the plants in the vegetative stage and direct nutrient resources toward leaf development, all the developing flowers were manually removed. The plants had uniform vigor and similar number of leaves (approximately 7–10 leaves) before the application of the fertilizer treatment.

### 2.2. Treatments

The experiment was conducted in a randomized complete block design (RCBD) with two treatments and three replications distributed across three blocks. ‘Yotsuboshi’ strawberry plants were assigned to one of two treatment groups: a control group (T1) receiving complete 14-14-14 NPK fertilizer or a nitrogen-deficient group (T2) receiving a 0-20-10 NPK formulation.

Fertilizer was applied on two occasions during the study, initially on 9 May 2025 (Day 0) and subsequently on 4 June 2025 (Day 26). To capture the rapid physiological effects of these quick-release fertilizers, which typically manifest within 5 to 7 days [31], leaf tissue samples were collected for analysis 6 days after each fertilizer application (Day 6 and Day 32). A final sample set was collected at the end of the 60-day observation period to verify that the distinct nitrogen levels established by the treatments were maintained throughout the experiment.

### 2.3. Nitrogen Quantification

**Figure 3:**
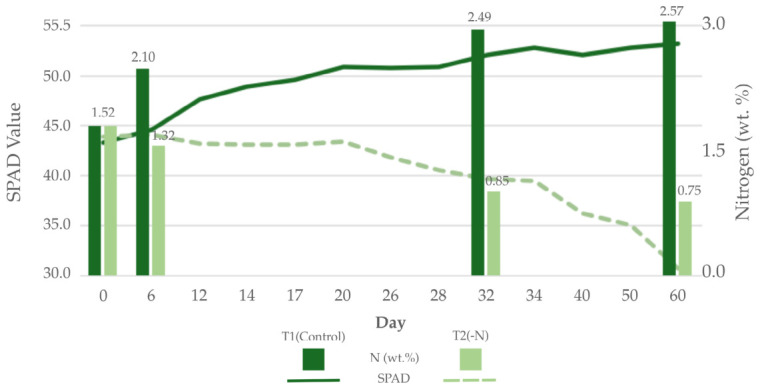
Temporal changes in SPAD values and leaf nitrogen content (wt%) of strawberry plants throughout the 60-day experiment.

To establish ground-truth data on plant nitrogen status, two quantitative measures were employed: nondestructive chlorophyll estimation using a SPAD meter and destructive leaf tissue analysis for total nitrogen content (wt%). SPAD readings were taken from each leaflet of three fully expanded leaves per replicate to noninvasively assess relative chlorophyll levels using a SPAD 502 (Minolta Ltd., Osaka, Japan), a portable spectrophotometer that enables rapid and nondestructive measurements of leaf greenness in the field (Figure 3) [32]. SPAD measurements are used to estimate the chlorophyll content, which is closely linked to the plant’s nitrogen level and provides insight into its photosynthetic activity [33]. In addition, for definitive nitrogen quantification, leaf tissue analysis was performed at multiple time points. An initial baseline was established by processing randomly collected leaf samples on Day 0. One trifoliate leaf per treatment in each block was subsequently collected on Days 6, 32, and 60. All the samples were dried, powdered, and submitted to the Chemical Analysis Division Research Facility Center for Science and Technology, University of Tsukuba Open Facility, for organic element analysis using an elementar UNICUBE instrument (Elementar Analysensysteme GmbH, Langenselbold, Germany) that utilizes the Dumas combustion method for analyzing nitrogen. The resulting nitrogen content is expressed as a weight percentage (wt%) for each collection date (Figure 3).

### 2.4. Data Collection and Storage

**Figure 4:**
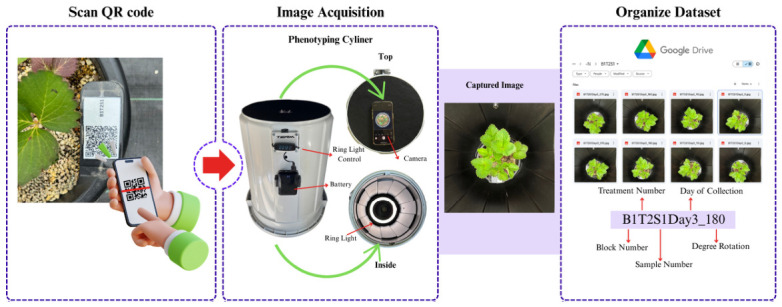
The QR coding system and phenotyping cylinder used to capture and organize the image dataset.

RGB images were acquired 13 times over the 60-day experimental period using a custom-built phenotyping cylinder (Figure 4). The apparatus was constructed from a 45-L bucket with a matte black interior to minimize reflections and external light interference. To ensure a controlled and consistent lighting environment, a 10-inch LED ring light powered by a portable battery was integrated into the design, effectively eliminating the environmental light variability typically found in field conditions. A precise aperture was placed at the top of the enclosure to accommodate a camera lens, allowing for the standardized collection of high-quality RGB imagery for nitrogen status detection. The study utilized a standard smartphone camera to capture RGB imagery, leveraging its widespread accessibility among the farming community. By using a device that most producers already own, the need for other specialized imaging equipment is eliminated, significantly lowering the barrier to adopting automated crop health monitoring solutions. RGB images were captured with the 0.5x Ultra-Wide camera of an iPhone 15 Pro Max (Apple, Cupertino, CA, USA) (13 mm, ƒ/2.2 aperture and 120° field of view, 100% Focus Pixels), set in a square aspect ratio, producing 3024 × 3024 pixel images. To obtain a comprehensive view of each plant, four images per replicate were taken by rotating the cylinder from 0° and at increments of 90°, 180°, and 270°, where the height between the plant canopy and the camera only varies approximately 10 cm (4 inches) during the observation period as the growth of strawberry plants is lateral rather than vertical. To ensure data consistency, all images were captured between 7:00 AM and 9:00 AM. This specific timeframe was selected to standardize physiological conditions across samples and further minimize the impact of environmental light variability before the peak solar intensity during the day. This imaging protocol resulted in 936 images, which were equally distributed between the control (T1, 468 images) and nitrogen-deficient (T2, 468 images) treatments.

To ensure systematic data organization and integrity for the deep learning dataset, a QR code-based management protocol was also established (Figure 4). Prior to fieldwork, a hierarchical folder structure was created in a cloud repository (Google Drive), where each folder’s file path was systematically named to embed key metadata: block, treatment, sample number, day of collection, and angle of capture. Unique QR codes, each encoding a hyperlink to one of these specific folders, were generated and physically affixed to their corresponding plant samples. The field data acquisition workflow involved scanning a sample’s QR code with a mobile device to automatically open the correct destination folder, into which the captured images were then directly saved. This integrated methodology automated data sorting at the point of collection, effectively mitigating manual labeling errors and ensuring a high-fidelity association between each image and its precise experimental metadata.

Consequently, multispectral images were acquired using a tripod-mounted Parrot^®^ Sequoia camera (Paris, France) at a height of 0.6 m on Days 6 and 32. This system captures four distinct spectral bands—green (550 nm), red (660 nm), red edge (735 nm), and near-infrared (NIR; 790 nm)—and includes a channel misalignment correction algorithm to ensure data accuracy at close range.

### 2.5. Data Processing

#### 2.5.1. RGB Images

A sufficient number of training images is recommended for the DL algorithms to learn better; thus, to increase the number of labeled datasets, the 936 images were augmented by horizontal and vertical flipping. These 2 augmentation procedures produced an additional 1872 images, resulting in a total training dataset of 2808 images. Image annotation for semantic segmentation was then performed using the open-source software LabelMe^®^ version 5.2.1. To ensure the creation of a robust, high-fidelity dataset and minimize labeling bias, a comprehensive, multicriteria protocol was established. This protocol required that each annotation be guided by the integration of four key criteria: (1) the assigned fertilizer treatment, (2) corresponding quantitative nitrogen data (SPAD values and nitrogen wt%), (3) the physiological age and position of the leaf on the plant, and (4) its distinct visual phenotype, including color and surface appearance.

1. Fertilizer Treatment

The assigned fertilizer treatment served as the primary criterion for the initial annotation of leaves (see Section 2.2 for details).

2. Quantitative Nitrogen Data (SPAD Value and Nitrogen wt%)

The quantitative ground-truth data, including SPAD values and tissue nitrogen content (N wt%), were used to objectively verify the labels. A nitrogen sufficiency threshold was established on the basis of the established literature, which defines the optimal range for strawberry leaves as 2.0% to 4.0% N [34]. On this basis, a definitive rule was applied: leaves whose measured nitrogen content was below the 2.0% threshold were assigned to the LowN class, while those whose content was above 2.0% were assigned to the NormalN class.

3. Physiological Age and Leaf Position

The integration of leaf age and position is critical for accurate nitrogen (N) diagnostics in strawberry plants. Research has demonstrated that nutrient concentrations fluctuate significantly across various phenological stages (age), suggesting that diagnostic baselines must be adjusted for physiological maturity to maintain accuracy [9].

**Figure 5:**
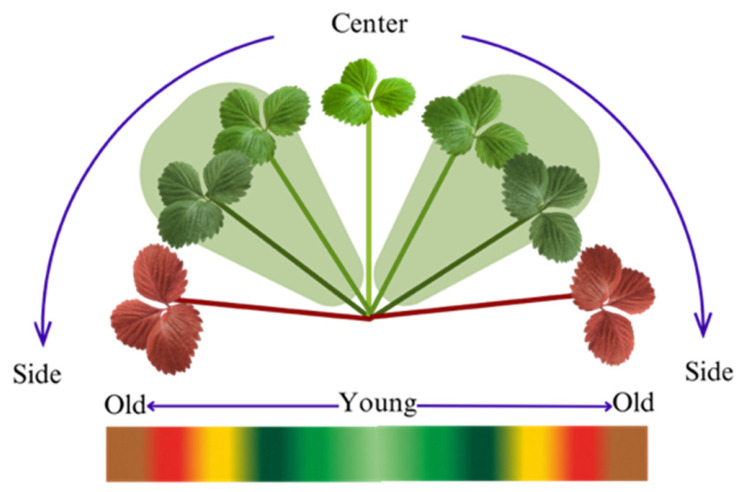
Physiological age, position and color changes of strawberry leaves that were considered for annotation.

To account for the confounding effects of leaf age, a strict selection criterion was implemented during the annotation process (Figure 5). Young, newly emerged leaves were explicitly excluded because their naturally lighter green pigmentation can be easily misidentified as deficiency-induced chlorosis. Furthermore, because nitrogen is a highly mobile nutrient, plants actively remobilize it from older, mature leaves (sources) to support the metabolic demands of new foliage (sinks) during periods of stress [6]. This physiological translocation causes nitrogen deficiency symptoms to manifest first and most severely on the older leaves located on the plant’s outer periphery. By focusing exclusively on these mature, outer leaves, our protocol effectively isolates the visual signals of nutrient stress from natural developmental color changes, ensuring a robust and physiologically grounded dataset for model training.

4. Color and Appearance of the Leaves

**Figure 6:**
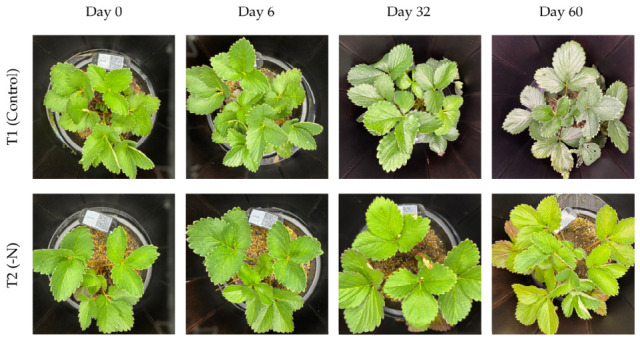
Temporal changes in the color and surface gloss of strawberry leaves under the experimental treatments.

The final assignment of labels was determined by the distinct visual phenotype of each selected mature leaf, considering both color and surface characteristics (Figure 6). A leaf was classified as a NormalN if it exhibited a uniform green color and a glossy surface. This glossiness is a key indicator, as it is determined by the protective cuticular wax layer of the leaf. The formation of this layer, like all metabolic processes in the plant, depends on overall plant health, for which an adequate supply of nitrogen is essential for the production of the necessary proteins and enzymes [35]. The LowN class was assigned to leaves displaying initial to moderate chlorosis, defined by a pale green to uniform yellow appearance. A third class, AdvancedLowN, was created to categorize leaves showing severe deficiency symptoms, characterized by the development of red pigmentation on the leaf margins or blades.

**Figure 7:**
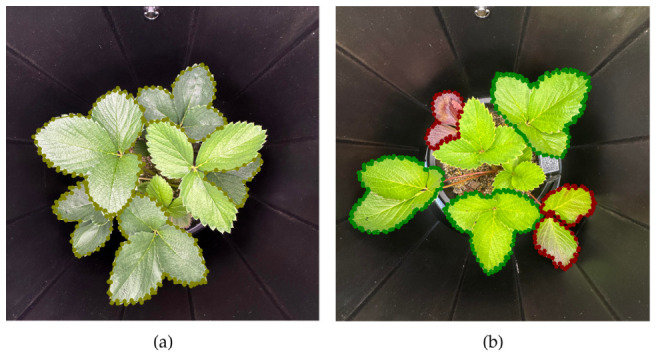
Example of an annotated image of the nitrogen status of strawberry leaves: (**a**) NormalN and (**b**) LowN (green mask) and AdvancedN (red mask).

On the basis of four predefined criteria, the image dataset was annotated using LabelMe^®^ software, with representative samples provided in Figure 7. In line with the trifoliate morphology of strawberry leaves, a single mask annotation was applied to each leaf, encompassing all three leaflets. Each annotation was initially saved as a JSON file containing the class labels and corresponding coordinates.

For compatibility with the You Only Look Once (YOLO) architecture, these JSON files were subsequently converted into the required TXT format. The complete dataset was then randomly partitioned into training and validation sets using an 80:20 split, resulting in 2246 and 562 images, respectively. A detailed breakdown of the annotation distribution across all classes is presented in Table 1.

#### 2.5.2. Multispectral Images

A total of 36 samples (T1(Control), 18; T2(-N), 18) from Days 6 and 32 were selected to calculate the normalized difference vegetation index (NDVI) using multispectral images captured by a Parrot^®^ Sequoia camera. To create the NDVI images, the near-infrared (NIR) and red band images were first aligned to correct for their spatial offset. This alignment was achieved by shifting the NIR image 80 pixels horizontally (along the *x*-axis) and 20 pixels vertically (along the *y*-axis) to match the red band image, which was used as the reference base. To calculate the average NDVI of the ‘Yotsuboshi’ strawberry plants, a multistep, image-processing approach was used to delineate the leaf regions from the background elements. Initially, a binary mask was generated by applying a global threshold to the NDVI image (NDVI > 0.2), which effectively isolated pixels corresponding to healthy vegetation. Subsequently, connected component analysis was performed on this binary mask to identify and label discrete, contiguous pixel clusters. To refine the segmentation and eliminate minor artifacts or noise, a size-based filtering step was implemented, discarding any components with an area below a 4500-pixel threshold. The final, consolidated mask, representing significant leaf areas of strawberry plants, was then used to selectively display the corresponding regions from a colorized NDVI map, thereby achieving clear visual segmentation of the foliage. This whole-plant approach was adopted because nitrogen is a mobile nutrient where the deficiency symptoms appearing on any single leaf are indicative of a systemic, plant-wide issue.

### 2.6. Training Process

**Figure 8:**
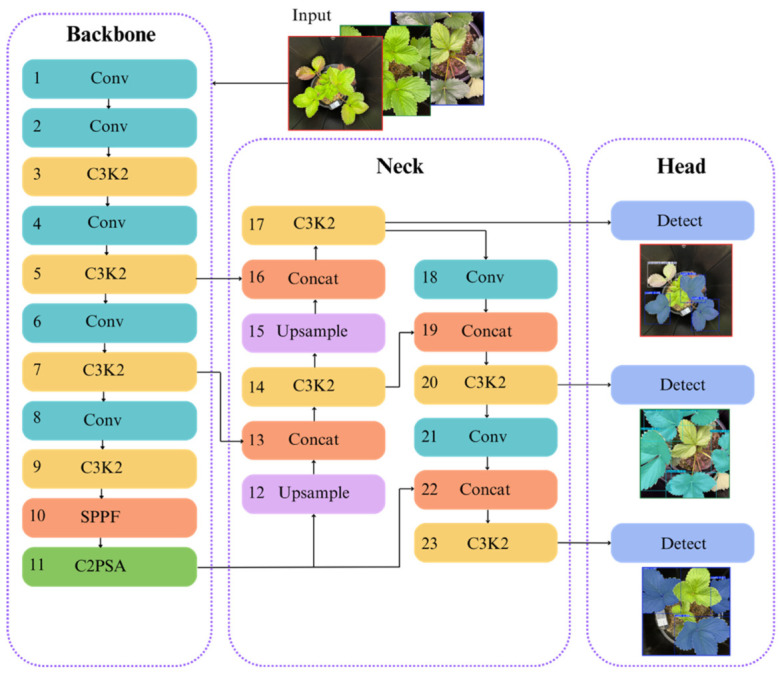
The structure of the YOLO11 network and detection.

For the object detection task in this study, the YOLO11 model was selected, representing one of the most advanced iterations in the YOLO series from Ultralytics. As a state-of-the-art model, YOLO11 brings faster processing, improved accuracy, and greater efficiency to computer vision tasks, supporting applications such as object detection, instance segmentation, and image classification [36]. Inheriting the high detection speed and end-to-end training characteristics of its predecessors, YOLO11 incorporates significant architectural optimizations to enhance performance (Figure 8).

Notably, the model integrates the C3k2 block, spatial pyramid pooling–fast (SPPF), and the convolutional block with parallel spatial attention (C2PSA) module. These components collectively improve feature extraction and object localization, significantly enhancing the detection and segmentation of objects across varied scales and orientations [36]. Such advancements result in superior accuracy and robustness, particularly in complex agricultural environments where both object localization and classification are challenging [37].

While newer iterations like YOLO12 have emerged, YOLO11 was prioritized for this research due to its superior reliability for field deployment. Unlike the stable release of YOLO11, YOLO12 remains a community-driven release that can exhibit training instability and elevated memory consumption [38]. Furthermore, the heavy attention blocks utilized in YOLO12 often lead to slower CPU throughput, which contradicts the requirements for lightweight, real-time agricultural applications of this study.

Consequently, YOLO11 marks a significant leap forward in real-time object detection, offering broader versatility and more dependable performance for diverse computer vision applications [38]. It is particularly instrumental for nitrogen deficiency detection as it consistently outperforms predecessors like YOLOv8 and YOLOv9 in recognizing subtle physiological patterns within complex agricultural backgrounds [39]. By achieving the optimal balance between high-precision accuracy, speed, background rejection, and computational efficiency, YOLO11 provides a robust framework for lightweight deployment in the real-time field monitoring of crop nitrogen status and the detection of early signs of plant stress [40]. This stability and optimization for both cloud environments and edge devices ensure that it can be smoothly incorporated into current agricultural operational workflows, fulfilling the practical requirements of this research [32,34,41].

The performance of the detection model was evaluated using standard object detection metrics, including precision, recall, and mean average precision (mAP). These metrics were calculated on the basis of the nitrogen status of the ‘Yotsuboshi’ strawberry leaves (NormalN, LowN, and AdvancedLowN) according to the equations below.

(1) $$\mathrm{Precision}=\frac{\mathrm{TP}}{\mathrm{TP}+\mathrm{FP}}$$

(2) $$\mathrm{Recall}=\frac{\mathrm{TP}}{\mathrm{TP}+\mathrm{FN}}$$

(3) $$\mathrm{MIoU}=\frac{\mathrm{Area}\;\mathrm{of}\;\mathrm{Overlap}}{\mathrm{Area}\;\mathrm{of}\;\mathrm{Union}}=\frac{\mathrm{TP}}{\mathrm{FP}+\mathrm{TP}+\mathrm{FN}}$$

- True Positives (TPs): The number of ‘Yotsuboshi’ strawberry leaves correctly identified and classified according to their nitrogen status.
- False Positives (FPs): The number of instances where the model incorrectly predicted a specific nitrogen status of the ‘Yotsuboshi’ strawberry leaves.
- False Negatives (FNs): The number of ‘Yotsuboshi’ strawberry leaves with a specific nitrogen status that the model failed to detect.

On the basis of these values, precision quantifies the accuracy of the model’s positive predictions of the nitrogen status of ‘Yotsuboshi’ strawberry, whereas recall measures its ability to identify all relevant instances within the dataset. The primary metric for overall model effectiveness was the mAP@50, which represents the mean average precision calculated at a 50% intersection over union (IoU) threshold. This score provides a comprehensive assessment of the model’s ability to both accurately localize and distinguish between the NormalN, LowN, and AdvancedLowN leaf classes.

## 3. Results

### 3.1. YOLO11 Training Results

The YOLO11 model in this research was trained using a hardware environment equipped with Windows 10, Intel(R) Core (TM) i7-10750H CPU @ 2.60 GHz, 32.0 GB of RAM, and an NVIDIA GeForce RTX 2060. The software environment included Anaconda 3, Python 3.7, and TensorFlow-GPU 1.13.1. The training configuration is presented in Table 2.

**Figure 9:**
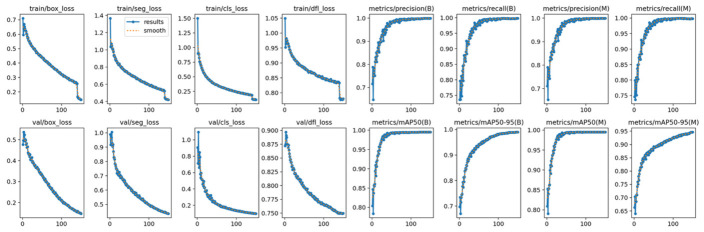
Training and validation results of YOLO11 in detecting the nitrogen status of ‘Yotsuboshi’ strawberry leaves.

This research utilized 2808 images for which the training and validation ratio was set to 80:20, comprising 2246 images for training and the remaining 562 for validation. The training results revealed that YOLO11 performed exceptionally well in detecting the nitrogen status of ‘Yotsuboshi’ strawberry leaves, with 99.9% precision, 99.8% recall and 99.5% mAP50 (Figure 9; Table 3).

**Figure 10:**
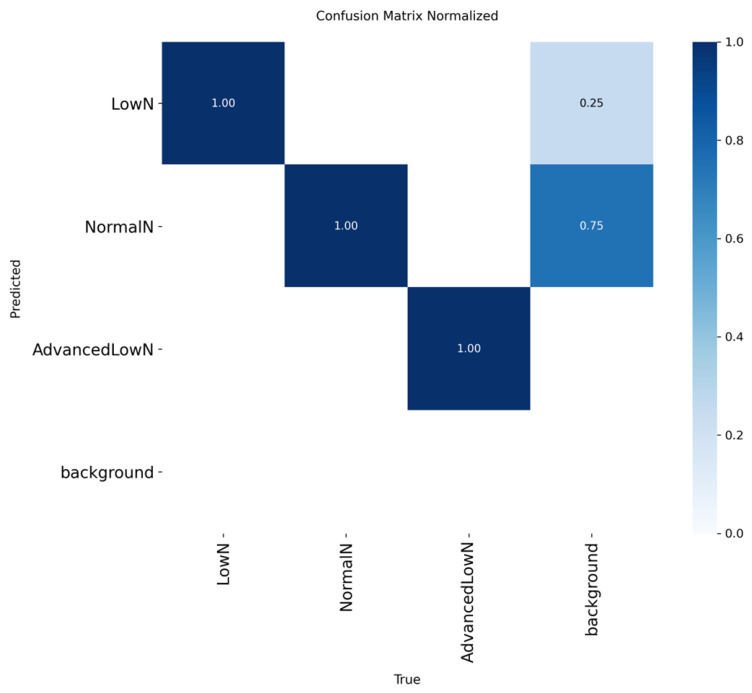
Normalized confusion matrix of the YOLO11 model for strawberry nitrogen status classification.

The normalized confusion matrix (Figure 10) demonstrates that the YOLO11 model achieved perfect classification accuracy (1.00) across all three nitrogen statuses, NormalN, LowN, and AdvancedLowN, indicating that the model can flawlessly distinguish between nutrient levels without inter-class error. The absence of off-diagonal misclassifications validates the effectiveness of the four-stage annotation protocol and the standardized lighting of the phenotyping cylinder in establishing distinct visual boundaries. While the model shows some tendency to predict background elements as leaves (0.75 for NormalN and 0.25 for LowN), its ability to correctly identify the physiological status of ‘Yotsuboshi’ strawberries remains highly robust.

### 3.2. YOLO11 Testing Results

- Test Image Dataset

The performance of the optimized YOLO11 model was evaluated on an independent test set of 30 images to assess its ability to detect three nitrogen status classes: NormalN, LowN, and AdvancedLowN. The evaluation yielded a precision of 82.5%, a recall of 92.5%, and a mean average precision at an IoU threshold of 0.5 (mAP@.50) of 87.2% (Table 4).

ii. Testing on standard wide-angle images

**Figure 11:**
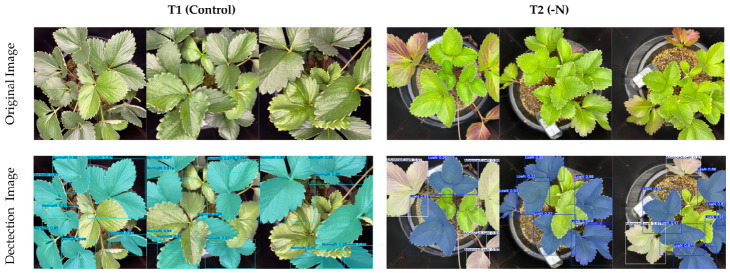
Test samples for the detection of the nitrogen status (NormalN-cyan, LowN-blue, AdvancedLowN-white) of ‘Yotsuboshi’ strawberry using standard wide-angle images.

Strawberry plants can grow and may differ in size during the growing season; to provide diverse testing images and simulate the growth of the plants, the model was further evaluated using 10 randomly selected images captured by a 1x zoom or 24 mm main camera and a standard wide-angle lens of the camera sensor (iPhone 15 Pro Max). The model achieved box mAP50 values of 86.7%, 84.5% precision and 76.3% recall and mask mAP50 values of 88.9%, 80.5% precision and 85.2% recall (Table 5). The sample and summary of the detection results are presented in Figure 11 and Table 6, respectively, which show 100% correct detection of the three-nitrogen status without negative or incorrect detections.

iii. Testing under open-field conditions

**Figure 12:**
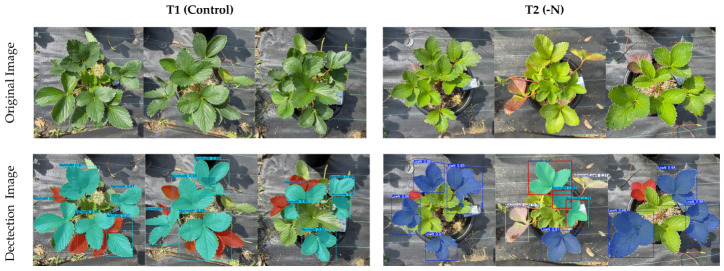
Test sample for the detection of nitrogen status in ‘Yotsuboshi’ strawberry using open-field (sunny) images. The cyan (NormalN), blue (LowN), white (AdvancedLowN) masks in the figure refer to True Positive (TP) or correct detection of the nitrogen status; the red mask signifies False Negative (FN) or undetected nitrogen status; and the red box indicates False Positive (FP) or incorrect detection.

**Figure 13:**
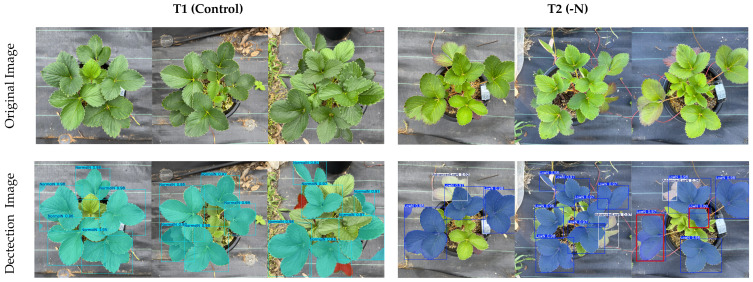
Test samples for the detection of nitrogen status in ‘Yotsuboshi’ strawberry using open-field (cloudy) images. The cyan (NormalN), blue (LowN), white (AdvancedLowN) masks in the figure refer to True Positive (TP) or correct detection of the nitrogen status; the red mask signifies False Negative (FN) or undetected nitrogen status; and the red box indicates False Positive (FP) or incorrect detection.

To assess the robustness of the model to variable illumination, a critical challenge for open-field applications, its performance was evaluated using images captured under two distinct open-field conditions. The first dataset was composed of images acquired under direct morning light, characterized by high-contrast lighting and strong shadows. The model achieved 85% precision, 69.3% recall and 79% mAP(50). The second dataset was captured under overcast conditions, representing a low-contrast environment with diffuse, uniform illumination, with 87.4% precision, 82.3% recall, and 82.7% mAP(50). The testing results of 10 images for both conditions are presented in Table 7 and Table 8. The sample and summary of the detection results are presented in Figure 12 and Figure 13 and Table 9 and Table 10, respectively, showing higher FN or undetected results in sunny than cloudy field conditions.

### 3.3. Comparative Analysis Through the NDVI

Comparing YOLO11 detection results with NDVI, despite having precise SPAD and nitrogen (N) data is essential for bridging the gap between point-specific leaf diagnostics and scalable field management. While SPAD and tissue analysis provide high-accuracy, contact-based physiological data, NDVI is the industry standard for the remote sensing tools commonly used in large-scale precision agriculture. Although NDVI can occasionally be less accurate than SPAD due to soil background interference or canopy saturation [42], its inclusion in this study serves as a critical calibration point to ensure that our deep learning model aligns with the metrics used by large-scale commercial farms.

Furthermore, leveraging this comparison supports the transition toward more accessible technology. For example, RGB indices captured by low-cost color cameras can effectively estimate complex parameters like NDVI, which typically require sophisticated and expensive equipment [43]. By confirming that our detection model, built on a rigorous four-stage annotation protocol (including SPAD and N), coincides with NDVI results, we validate our methodology as a robust and cost-effective alternative. This cross-validation ensures that our system provides the same diagnostic fidelity as high-end sensors while remaining feasible for small to medium-scale operations.

**Figure 14:**
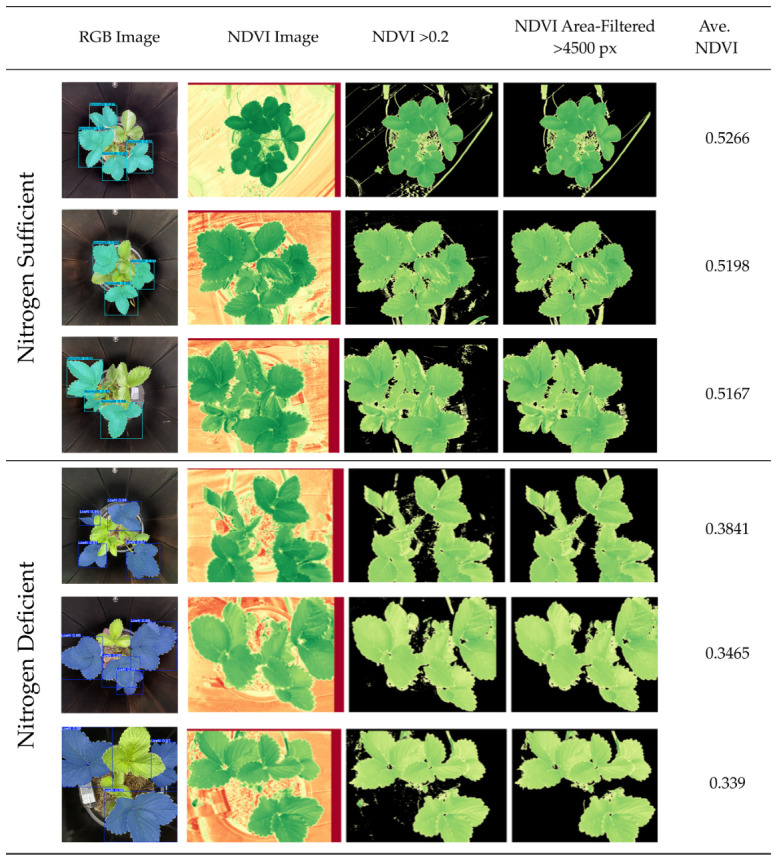
Sample-processed normalized difference vegetation index (NDVI) images. This process produced the images and the average NDVI value for each plant sample.

To validate the performance of the proposed RGB-based deep learning model, the detection results were compared to the 36 NDVI images and values under control (T1) and nitrogen-deficient (T2) conditions on Days 6 and 32 (Figure 14; Table 11). At the initial measurement on Day 6, a pronounced and statistically significant difference in the NDVI was observed, with the T1 control group exhibiting a mean NDVI of 0.521 and the T2 nitrogen-deficient group showing a much lower mean NDVI of 0.366. Over the subsequent 26 days, the two groups followed divergent developmental paths. The mean NDVI of the T1 control group increased to 0.533, indicating healthy plant development and biomass accumulation. In contrast, the mean NDVI of the T2 nitrogen-deficient group decreased slightly to 0.354.

The results provide unequivocal evidence of the critical role of nitrogen in plant health, as quantified by the NDVI. A substantial difference in the NDVI values was apparent from the first measurement on Day 6, indicating that nitrogen deficiency imposed immediate and severe physiological stress. A critical finding was the divergence of the NDVI trajectories over time; the control group’s NDVI increased, indicating normal growth, whereas the nitrogen-deficient group’s NDVI decreased, indicating the progressive impact of nutrient stress.

## 4. Discussion

Conventional nutrient assessment methods, like laboratory tissue analysis, are inherently destructive, costly, and slow for real-time management. While SPAD meters and AUVs offer nondestructive alternatives, they lack the integrated simplicity and scalability required for scalable, immediate, in-field decision-making. This study presents a fast, cost-effective, and nondestructive method for determining nitrogen status in a new Japanese strawberry variety, ‘Yotsuboshi’, by leveraging deep learning technology, providing an accurate decision-support tool for precision nutrient management.

Our methodology advances precision agriculture by integrating a custom phenotyping cylinder with deep learning to bridge the gap between lab-level accuracy and field practicality. By providing a standardized light environment, the cylinder eliminates environmental light variability, allowing models like YOLO11 to reliably detect nitrogen indicators. This approach replaces expensive sensors with accessible smartphone-based RGB imagery, offering a low-cost, variety-specific solution for the high-density cultivation of ‘Yotsuboshi’ strawberries. Ultimately, this combination enables a scalable “sense-and-act” framework suitable for real-time, automated crop monitoring.

### 4.1. Data Collection, Storage and Processing

To mitigate the primary obstacle of unstable ambient lighting in field-based detection, this study utilized a custom-built phenotyping cylinder with integrated lighting to standardize image acquisition and a QR code system for error-free data management. The resulting dataset was validated through a rigorous four-stage annotation protocol: (1) fertilizer treatments, (2) SPAD and tissue analysis, (3) targeted sampling of mature leaves, and (4) visual phenotyping. This comprehensive approach ensured that the model was trained on annotations anchored in quantitative physiological evidence, providing a robust foundation for the accurate detection of nitrogen status in ‘Yotsuboshi’ strawberries.

### 4.2. Performance Analysis of YOLO11

The YOLO11 architecture demonstrated exceptional capability in detecting ‘Yotsuboshi’ strawberry nitrogen status, achieving a test mAP50 of 87.2%. This performance significantly surpasses previous ResNet-50 benchmarks (78%) [26] and is on par with the performance of CNN (87%) in detecting nitrogen deficiency in sorghum under field conditions [44]. These results confirm that nitrogen deficiency phenotypes are distinct and highly learnable when using advanced object detection frameworks.

However, the model’s robustness is heavily influenced by the interaction between the glossy surface of strawberry leaves and fluctuating environmental light. While standardized imaging yielded an 88.9% mAP50, direct sunlight caused reflections and pixel saturation, dropping accuracy to 79% [45]. In contrast, diffuse (cloudy) lighting improved performance to 82.7% by mimicking the uniform distribution of the custom phenotyping cylinder. These findings identify inconsistent lighting, rather than background complexity, as the primary obstacle for field deployment, highlighting the importance of utilizing standardized acquisition tools such as phenotyping cylinders in the open-field detection of nitrogen status in strawberry to preserve biological signals against technical noise.

### 4.3. Comparison of the YOLO11 and NDVI Results

Our strategy offers a streamlined, computationally efficient alternative to traditional NDVI estimation from RGB data [46], making it highly accessible for small to medium-scale strawberry farms. The predictive accuracy of the YOLO11 model was strongly validated by ground-truth NDVI data, as ‘NormalN’ and ‘LowN’ classifications aligned precisely with physiological growth trajectories. This direct correlation demonstrates that our deep learning framework can be an effective alternative to complex NDVI methods while maintaining high diagnostic fidelity, providing a robust and practical tool for real-time, nondestructive nitrogen monitoring in ‘Yotsuboshi’ strawberry.

## 5. Conclusions

This research proposed a methodology for the nondestructive determination of nitrogen status in ‘Yotsuboshi’ strawberries under field conditions by integrating RGB image analysis with the YOLO11 deep learning algorithm and comparative NDVI analysis. This approach was designed to provide a low-cost, high-throughput diagnostic solution that is accessible to small- and medium-sized strawberry growers. Some of the key contributions of this research are outlined below:

(1) A low-cost phenotyping cylinder was developed to mitigate the effects of ambient light variability, which is a common issue in field-based research, and standardize illumination for consistent, nondestructive data collection. A novel dataset was subsequently created using this apparatus for training and monitoring the nitrogen status of ‘Yotsuboshi’ strawberry.
(2) A rigorous four-stage annotation criterion was implemented to generate a high-quality dataset for detecting the nitrogen status of ‘Yotsuboshi’ strawberry plants. The robustness of the dataset directly enabled the superior training performance of the YOLO11 model, as evidenced by a precision of 99.9% and an mAP50 of 99.5%.
(3) A streamlined methodology was introduced for nitrogen status detection in ‘Yotsuboshi’ strawberry plants. This research offers a more accessible diagnostic tool by replacing conventional NDVI analysis, which requires multispectral imagery (red and NIR bands), with a deep learning model (YOLO11) that operates on standard RGB images. This simplification of the data acquisition and analysis pipeline provides a method for nitrogen status diagnosis that is both rapid and highly accurate. The efficacy of this rapid diagnostic method was confirmed by validating the model’s ‘NormalN’ and ‘LowN’ classifications against empirical NDVI data, which demonstrated high accuracy without the need for more complex imaging techniques.

This research also identified the primary challenge for real-world applications: variable ambient lighting. While the model performed robustly in controlled settings and under diffuse cloudy conditions (mAP50 of 82.7%), its accuracy decreased significantly in direct sunlight (mAP50 of 79%). This highlights that inconsistent illumination, which causes harsh shadows and highlights, is a more critical obstacle than background complexity for in-field deployment. Thus, the utilization of a phenotyping cylinder in field detection is essential for providing accurate nitrogen status results for ‘Yotsuboshi’ strawberry. Ultimately, integrating this technology into a mobile application or robotic platform could revolutionize real-time nutrient management, leading to more efficient, cost-effective, and sustainable farming practices.

## Figures and Tables

**Table 1:** Summary of the distribution of annotations for each class.

Set	Class			Total Annotation	Images
	NormalN	LowN	AdvancedLowN		
Training	5177	4909	975	11,061	2246
Validation	1324	1111	281	2716	562
Total	6501	6020	1256	13,777	2806

**Table 2:** Training configuration for the YOLO11 model.

Parameters	Values
Input Size	640 × 640
Batch Size	4
Epochs	150

**Table 3:** YOLO11 model training performance in detecting the nitrogen status of ‘Yotsuboshi’ strawberry leaves.

Parameters	Box	Mask
Precision	0.999	0.999
Recall	0.998	0.998
mAP50	0.995	0.995
mAP50-95	0.99	0.947

**Table 4:** YOLO11 model performance in detecting the nitrogen status of ‘Yotsuboshi’ strawberry leaves.

Annotation	Instances	Box			Mask		
		Precision	Recall	mAP(50)	Precision	Recall	mAP(50)
NormalN	72	0.78	0.926	0.834	0.78	0.926	0.834
LowN	50	0.785	1	0.861	0.785	1	0.861
AdvancedLowN	29	0.911	0.85	0.921	0.911	0.85	0.921
ALL	151	0.825	0.925	0.872	0.825	0.925	0.872

**Table 5:** YOLO11 model performance in detecting the nitrogen status of ‘Yotsuboshi’ strawberry leaves using standard wide-angle images.

Annotation	Instances	Box			Mask		
		Precision	Recall	mAP(50)	Precision	Recall	mAP(50)
NormalN	37	0.839	0.701	0.808	0.794	0.757	0.856
LowN	20	0.817	0.671	0.822	0.712	0.8	0.822
AdvancedLowN	12	0.879	0.917	0.971	0.91	1	0.989
ALL	69	0.845	0.763	0.867	0.805	0.852	0.889

**Table 6:** Summary of the detection results of the YOLO11 model on the standard wide-angle sample images.

Label	True Positive	False Negative	False Positive
NormalN	17	0	0
LowN	14	0	0
AdvancedLowN	5	0	0

**Table 7:** Testing performance for the detection of nitrogen status in ‘Yotsuboshi’ strawberry under open-field (sunny) conditions using YOLO11.

Annotation	Instances	Box			Mask		
		Precision	Recall	mAP(50)	Precision	Recall	mAP(50)
NormalN	41	0.675	0.658	0.698	0.675	0.658	0.709
LowN	28	0.878	0.679	0.83	0.878	0.679	0.83
AdvancedLowN	12	1	0.744	0.829	1	0.744	0.829
ALL	81	0.851	0.693	0.786	0.851	0.693	0.789

**Table 8:** Testing performance for the detection of nitrogen status in ‘Yotsuboshi’ strawberry under open-field (cloudy) conditions using YOLO11.

Annotation	Instances	Box			Mask		
		Precision	Recall	mAP(50)	Precision	Recall	mAP(50)
NormalN	20	1	0.823	0.884	1	0.823	0.884
LowN	30	0.835	0.845	0.859	0.835	0.845	0.859
AdvancedLowN	5	0.787	0.8	0.738	0.787	0.8	0.738
ALL	55	0.874	0.823	0.827	0.874	0.823	0.827

**Table 9:** Summary of the results of nitrogen status detection in ‘Yotsuboshi’ strawberry plants using the YOLO11 model. All sample images were captured under open-field conditions (sunny).

Label	True Positive	False Negative	False Positive
NormalN	13	9	0
LowN	8	0	3
AdvancedLowN	2	2	0

**Table 10:** Summary of the detection results of nitrogen status in ‘Yotsuboshi’ strawberry plants using the YOLO11 model. All sample images were captured under open-field conditions (cloudy).

Label	True Positive	False Negative	False Positive
NormalN	16	2	0
LowN	13	0	1
AdvancedLowN	3	0	1

**Table 11:** Summary of Normalized Difference Vegetation Index (NDVI) values of sample images from Day 6 and Day 32.

Day of Observation	Treatment	Mean NDVI	Std. Dev. (SD)	*t*-Statistic	*p*-Value
Day 6	T1 (Control)	0.521	0.008	19.53	<0.0001
	T2 (-N)	0.366	0.02		
Day 32	T1 (Control)	0.533	0.008	20.97	<0.0001
	T2 (-N)	0.354	0.022		

## Data Availability

The dataset that was generated and used in this study is available upon request from the corresponding author, but restrictions apply to data reproducibility and commercially confidential details.
